# Effect of cilostazol on arterial stiffness and vascular adhesion molecules in type 2 diabetic patients with metabolic syndrome: a randomised, double-blind, placebo-controlled, crossover trial

**DOI:** 10.1186/1758-5996-5-41

**Published:** 2013-07-26

**Authors:** Nam Hoon Kim, Hee Young Kim, Hyonggin An, Ji A Seo, Nan Hee Kim, Kyung Mook Choi, Sei Hyun Baik, Dong Seop Choi, Sin Gon Kim

**Affiliations:** 1 Division of Endocrinology and Metabolism, Department of Internal Medicine, Korea University Anam Hospital, Korea University College of Medicine, 126-1, Anam-dong 5-ga, Seongbuk-gu, Seoul 136-705, Korea; 2 Department of Biostatistics, Korea University College of Medicine, Seoul, Korea

**Keywords:** Cilostazol, Phosphodiesterase inhibitor, Arterial stiffness, Vascular adhesion molecules, Type 2 diabetes, Metabolic syndrome

## Abstract

**Background:**

The phosphodiesterase inhibitor cilostazol has beneficial effects on atherosclerosis by virtue of vasodilatory and antiplatelet effects. However, less is known about the effect of cilostazol on arterial stiffness and biochemical markers related to vascular inflammation and endothelial dysfunction in type 2 diabetic patients with metabolic syndrome.

**Methods:**

In this randomized, double-blind, crossover trial, 45 diabetic patients with metabolic syndrome were randomly assigned to either the cilostazol group (50 mg for 2 weeks, 100 mg for 6 weeks) or placebo group for an 8-week treatment phase, and then crossed over. Brachial-ankle pulse wave velocity (baPWV) and serum levels of inflammatory cytokines and vascular cellular adhesion molecules were measured before and after each treatment phase.

**Results:**

Compared with the placebo group, the mean baPWV did not improve in the cilostazol group (mean difference 31.42 cm/sec, 95% CI −55.67 to 118.5). Cilostazol treatment significantly reduced soluble vascular cellular adhesion molecule-1 (sVCAM-1) level (from 1288.7 ± 285.6 to 1168.2 ± 252.3 ng/dL, *P* = 0.0003), and there was also significant mean difference between groups (mean difference 105.18 ng/dL, 95% CI 10.65 to 199.71). However, other biochemical markers including lipid profiles, high sensitivity C-reactive protein, adiponectin, interleukin-6, tumor necrosis factor-alpha, monocyte chemotactic protein-1, and soluble intercellular adhesion molecule-1 did not improve with cilostazol treatment.

**Conclusion:**

Cilostazol treatment significantly reduced serum sVCAM-1 level, but this short term treatment was not associated with beneficial effect on arterial stiffness and other inflammatory markers.

**Trial registration:**

(Clinical trial reg. no. NCT00573950, clinicaltrials.gov.)

## Introduction

Cardiovascular disease (CVD) is the major cause of mortality in patients with diabetes and metabolic syndrome. Chronic inflammation and endothelial dysfunction appear to be major components contributing to the development of atherosclerosis in a background of insulin resistance in diabetic patients [1,2]. Biochemical markers such as proinflammatory cytokines and vascular cellular adhesion molecules are related to the progression of atherosclerosis and CVD [3,4] and are also helpful for the screening of subclinical atherosclerosis [5,6].

On the other hands, subclinical atherosclerosis and increased arterial stiffness are early changes predicting the development of CVD. Various noninvasive measures of these changes, such as carotid intima-media thickness (IMT), pulse wave velocity (PWV), and coronary artery calcification have been developed and commonly used [7]. Of these methods, PWV is the most commonly used measure of arterial stiffness, and predicts the development of CVD and mortality in type 2 diabetes [8].

Cilostazol is a selective inhibitor of cyclic phosphodiesterase 3, which is used for the treatment of chronic peripheral artery disease, by virtue of its antiplatelet and vasodilatory activities [9]. A growing body of evidence obtained from animal models suggests that cilostazol has beneficial effects on lipid metabolism either by cAMP-mediated increases in lipoprotein lipase, or by inhibiting the production of cytokines related to the progression of atherosclerotic lesions [10,11].

Previous clinical studies showed that cilostazol treatment attenuated the progression of carotid IMT in type 2 diabetic patients [12,13]. The favorable effects of cilostazol on atherosclerosis were also demonstrable in diabetic patients with known peripheral artery disease (PAD); soluble CD40 ligand and hs-CRP levels were significantly decreased, and arterial compliance was modestly improved [14,15], However, the effects on arterial stiffness and various inflammatory markers related to the progression of atherosclerosis have not been evaluated in subjects with type 2 diabetes but not having coronary heart disease or PAD. Therefore, we conducted a randomized, double-blind, crossover study to clarify the efficacy of cilostazol in preventing the progression of arterial stiffness using brachial-ankle PWV (baPWV) in patients with type 2 diabetes and metabolic syndrome. We also evaluated the effect of cilostazol on proinflammatory cytokines and inflammatory markers related to vascular inflammation and endothelial dysfunction.

## Materials and methods

### Subjects and study design

The eligible participants were patients aged over 18 years with type 2 diabetes and metabolic syndrome. Type 2 diabetes was defined by the American Diabetes Association criteria [16]. All patients had been treated with oral hypoglycemic agents or insulin. Metabolic syndrome was defined according to Adult Treatment Panel III of the National Cholesterol Education Program guidelines [17], modified in accordance with the World Health Organization’s proposed waist circumference (WC) cut-off points for Asians. Subjects with metabolic syndrome were required to meet three or more of the following criteria: WC ≥90 cm in men and ≥80 cm in women, serum triglyceride level ≥150 mg/dL, HDL-cholesterol (HDL-C) levels <40 mg/dL in men and <50 mg/dL in women, impaired fasting glucose ≥100 mg/dL or anti-diabetic treatment, and blood pressure ≥130/85 mmHg or treatment for hypertension.

Subjects who had taken a stable dose of anti-hypertensive or lipid-lowering drugs for at least 8 weeks before the study were enrolled, and these medication dosages were maintained throughout the study. The major exclusion criteria included known CVDs including coronary heart disease and peripheral artery disease; congestive heart failure; severe hepatic dysfunction and renal dysfunction; treatment with thiazolidinendione within 8 weeks at screening; current use of anticoagulants, antiplatelet agents, and corticosteroid supplements; and history of drug or alcohol abuse. All subjects gave informed consent and the protocol was approved by the Institutional Review Board of Korea University Hospital.

Of 49 Korean subjects screened, 4 did not meet the diagnostic criteria of metabolic syndrome. As a result, 45 Korean patients were randomly assigned to receive cilostazol (50 mg for the first 2 weeks and 100 mg for the next 6 weeks) or matching placebo for 8 weeks. The dose titration was set to minimize adverse events related to study medications.

With a washout period of 8 weeks, participants were then crossed over to the other treatment arm for a further 8 weeks. Cilostazol and placebo tablets were supplied by Otsuka Pharmaceuticals (Tokyo, Japan). The tablets were identical in size, color, and taste. All participants underwent a comprehensive physical examination, anthropometric measurements, and blood sampling for biochemical analyses, and baPWV measurements at baseline and after treatment at each treatment phase. Blood pressure was measured in a standardized manner with a mercury sphyngomanometer; two separate measurements were taken after 5 min of rest and the mean was calculated. Anthropometric measurements were taken after an overnight fast. Height, body weight, BMI (kg/m^2^), and WC of all subjects were recorded. Blood was drawn for biochemical analysis after an overnight fast. This clinical trial is registered as NCT00573950 at clinicaltrials.gov.

### Measurement of arterial stiffness

Arterial stiffness was assessed by baPWV, which was measured using a model BP-203RPE II volume-plethymographic apparatus (Colin, Komaki, Japan). Each participant was examined in a supine position with electrocardiographic electrodes placed on both wrists and cuffs wrapped around both brachia and ankles. The pulse waveforms were recorded using a semiconductor pressure sensor. Transmission time was calculated as the time for the waveform to travel between the right arm and both ankles, and the transmission distance between the right brachium and ankle was automatically calculated based on the height of the participant. The baPWV was automatically calculated by dividing transmission distance by the transmission time. The means of right and left baPWV were used for the analysis.

### Measurement of biochemical markers

Venous blood samples were drawn from each patient after overnight fasting. Blood samples were centrifuged to obtain serum, and the serum was stored at −80°C. High sensitivity C-reactive protein (hsCRP) levels were measured using a CRP ELISA kit (Immunodiagno, Bensheim, Germany); the intra-and interassay coefficients of variation were 6.5% and 10.4%, respectively. Total cholesterol, triglyceride, HDL-C, and LDL-C levels were determined by enzymatic methods using a model 747 automated clinical chemistry analyzer (Hitachi, Tokyo, Japan). Homeostasis model assessment of insulin resistance index was calculated using the the formula (fasting insulin [μIU/mL] × fasting glucose [mmol/L] / 22.5), and quantitative insulin sensitivity check index was calculated as 1 / (log fasting insulin [μIU/mL] + log fasting glucose [mg/dL]).

Serum levels of adiponectin, interleukin-6 (IL-6), soluble vascular cellular adhesion molecule-1 (sVCAM-1), soluble intercellular adhesion molecule-1 (sICAM-1), monocyte chemotactic protein-1 (MCP-1), and tumor necrosis factor-alpha (TNF-α) were measured using a MILLIPLEX™ Human Cytokine/Chemokine panel (Millipore, Billerica, MA). Intra- and interassay coefficients of variation were 9.2 and 15.9% for adiponectin, 8.1 and 11.6% for IL-6, 4.5 and 8.5% for sVCAM-1, 7.9 and 9.7% for sICAM-1, 6.1 and 12.0% for MCP-1, and 10.5 and 15.9% for TNF-α, respectively.

### Statistical analysis

SAS PROC MIXED (SAS Institute, Cary, NC) was used to analyze the data from the crossover trial. The Grizzles model included treatment effect, carry-over effect, and sequential effect as fixed effects and the subject effect as a random effect. The model was fitted for seven different responses (baPWV, IL-6, TNF-α, adiponectin, MCP-1, sVCAM-1, and sICAM-1). There were no significant carry-over and sequential effects. Baseline characteristics of study participants were comparable in both groups and both phases. Primary outcome was change in arterial stiffness measured by baPWV. Secondary outcome was change in inflammatory markers. The sample size of the study was determined on the basis of the estimation for the primary outcome of baPWV from previous trials [18,19]. Using a two-sided test for differences in independent binomial proportions with an alpha level of 0.05, 36 patients would have to undergo randomization for the study to have 80% power to detect a difference in the baPWV between the two study arms. Therefore, at least 48 patients were enrolled to account for an anticipated 25% loss in follow-up. The level of significance was 0.05. All analyses were performed using SAS version 9.1.3 (SAS Institute).

## Results

**Figure 1:**
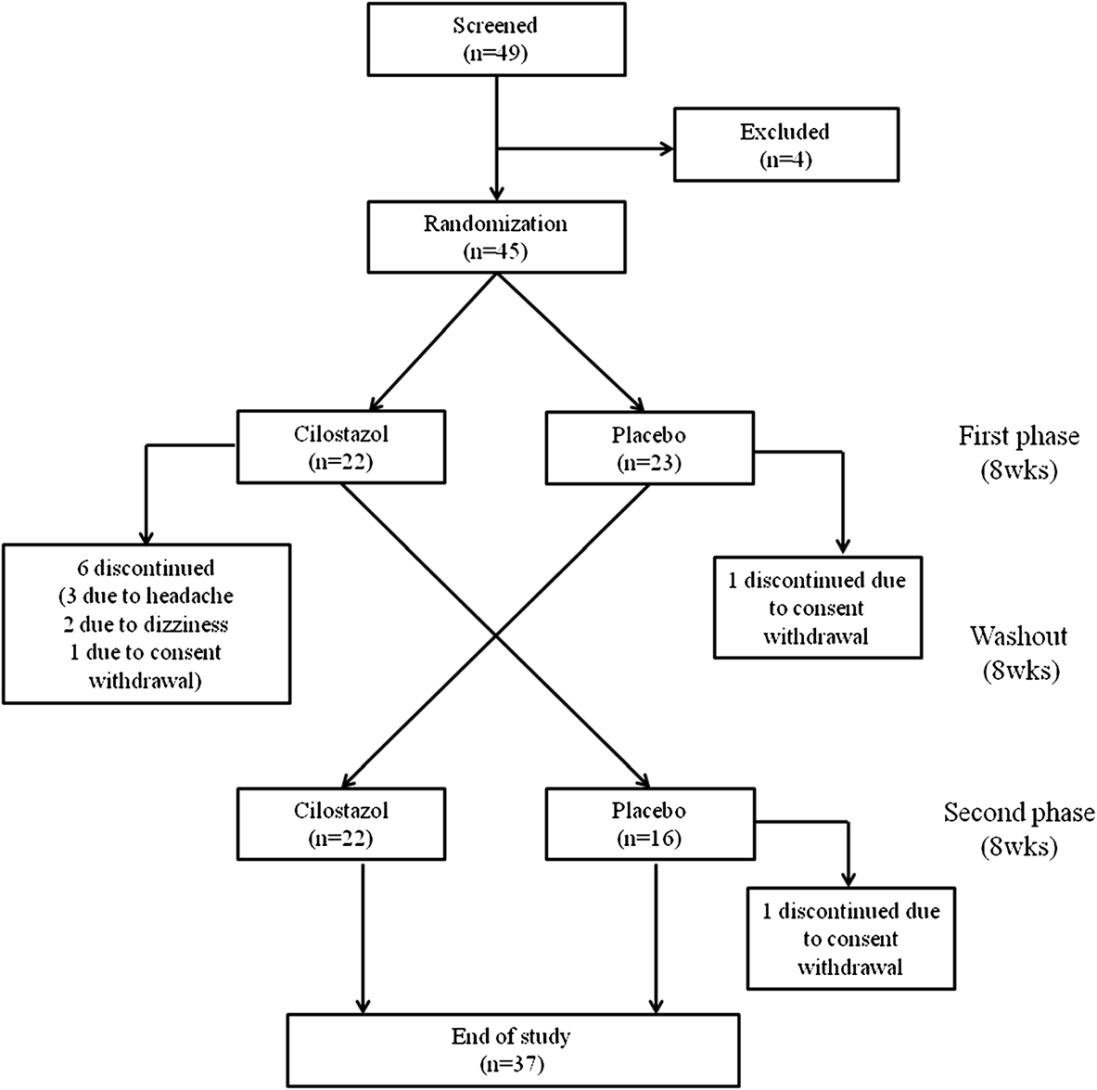
Trial design.

Forty five participants were randomly assigned to each study arm. Two participants withdrew their consent for participation before the first treatment phase and one participant withdrew during the washout period. During the first phase, two patients in the cilostazol arm withdrew due to headache (*n* = 1) and dizziness (*n* = 1). After completion of cilostazol treatment, three patients withdrew due to headache (*n* = 2) and dizziness (*n* = 1). As a result, 37 participants completed the full protocol (Figure 1). Throughout the study period, the medications were generally well tolerated by the 37 subjects. One participant experienced mild headaches but completed the study.

Table 1 summarizes the baseline characteristics of the participants. The subjects comprised 15 males (40.5%) and the mean age of the total participants was 61.2 ± 6.7 years. The mean duration of diabetes was 10 ± 6 years and the mean BMI was 26.2 ± 2.9 kg/m^2^. The anthropometric and biochemical data showed that, for most participants, diabetes was generally well-controlled. The mean glycated hemoglobin (HbA1c) level was 7.1%, mean systolic and diastolic pressure was 131.9 and 79.2 mmHg, respectively, mean total cholesterol was 155.2 mg/dL, and mean LDL cholesterol was 91.6 mg/dL.

**Table 1:** Baseline charateristics of participants (n = 37)

**Characteristic**	**Mean ± SD (or N/%)**
Age (years)	61.2 ± 6.7
Male sex (n/%)	15 (40.5%)
Duration of diabetes (years)	10 ± 6
Hypertension	32 (86.5%)
Dyslipidemia	14 (37.8%)
Current smoker	13 (35.1%)
BMI (kg/m^2^)	26.2 ± 2.9
Waist circumference (cm)	92.5 ± 8.0
Systolic blood pressure (mmHg)	131.9 ± 11.8
Diastolic blood pressure (mmHg)	79.2 ± 11.4
Fasting glucose (mg/dL)	133.4 ± 27.1
HbA1c (%)^*^	7.1 ± 1.1
Fasting insulin (μIU/mL)^*^	11.6 ± 1.6
HOMA-IR^*^	3.8 ± 1.6
QUICKI	0.32 ± 0.02
Total cholesterol (mg/dL)	155.2 ± 25.2
Triglyceride (mg/dL)^*^	131.1 ± 1.7
HDL-C (mg/dL)	46.2 ± 9.9
LDL-C (mg/dL)	91.6 ± 21.2
hsCRP (mg/L)^*^	1.07 ± 2.65
Mean baPWV (cm/sec)	1591.4 ± 192.6
Adiponectin (ng/mL)^*^	8.86 ± 1.87
IL-6 (pg/mL)^*^	3.99 ± 2.94
TNF-α (ng/dL)^*^	6.36 ± 1.27
MCP-1 (ng/dL)^*^	590.2 ± 1.63
sVCAM-1 (ng/dL)	1224.4 ± 267.6
sICAM-1 (ng/dL)^*^	169.7 ± 1.29

*HOMA-IR*, homeostasis model assessment of insulin resistance, *QUICKI* quantitative insulin sensitivity check index, *hsCRP* high sensitivity C-reactive protein, *baPWV* brachial-ankle pulse wave velocity, *IL-6* interleukin-6, *TNF-α* tumor necrosis factor-alpha, *MCP-1* monocyte chemotactic protein-1, *sVCAM-1* soluble vascular cellular adhesion molecule-1, *sICAM-1* soluble intercellular adhesion molecule-1.

Data are presented as the mean ± SD. ^*^Geometric mean ± SD.

Table 2 shows changes from baseline to week 8 of anthropometric and metabolic parameters in both comparison groups. Baseline parameters were not significantly different in both groups. After the 8-week treatment with cilostazol or placebo, no anthropometric measure had changed substantially. However, WC in the cilostazol group significantly decreased from 92.6 to 91.4 cm (*P* = 0.007). Lipid profiles including total cholesterol, triglyceride, and HDL-C improved in both groups, but none of the improvements was statistically significant. Cilostazol treatment lowered mean baPWV from 1621.8 ± 229.8 to 1592.0 ± 249.3 cm/sec, but the decrease was not statistically significant. Most of inflammatory markers including adiponectin, IL-6, MCP-1, sVCAM-1, and sICAM-1 showed tendency of improvement with cilostazol. However, there was significant change only in sVCAM-1 level (1288.7 ± 285.6 to 1168.2 ± 252.3 ng/dL, *P* = 0.0003) and a modest change in serum adiponectin level (9.17 ± 1.88 to 9.92 ± 1.93 ng/mL, *P* = 0.07). There were no significant changes of outcomes in the placebo group except IL-6 (4.13 ± 3.14 to 3.21 ± 3.34 pg/mL, *P* = 0.02).

**Table 2:** Changes from baseline to week 8 of anthropometric and metabolic parameters in cilostazol and placebo group (n=37)

	**Placebo**			**Cilostazol**		
	**Baseline**	**Follow-up**	***P*-value**	**Baseline**	**Follow-up**	***P*-value**
BMI (kg/m^2^)	26.2 ± 2.9	26.2 ± 2.9	0.29	26.2 ± 2.9	26.0 ± 2.9	0.12
Waist circumference (cm)	92.5 ± 8.0	92.1 ± 7.6	0.36	92.6 ± 8.0	91.4 ± 7.4	0.007
Systolic blood pressure (mmHg)	131.0 ± 10.8	131.1 ± 12.0	0.93	130.6 ± 11.2	129.6 ± 13.0	0.59
Diastolic blood pressure (mmHg)	79.6 ± 11.2	81.3 ± 10.3	0.35	79.4 ± 9.8	77.4 ± 9.3	0.20
Fasting glucose (mg/dL)	129.2 ± 22.2	129.6 ± 32.0	0.94	137.5 ± 41.2	140.2 ± 31.7	0.66
HbA1c (%)^*^	7.2 ± 1.2	7.1 ± 1.2	0.19	7.3 ± 1.2	7.2 ± 1.2	0.42
Fasting insulin (μIU/mL)^*^	11.0 ± 1.6	11.0 ± 1.6	0.99	11.1 ± 1.5	10.3 ± 1.5	0.21
HOMA-IR^*^	3.5 ± 1.6	3.6 ± 1.7	0.50	3.6 ± 1.6	3.5 ± 1.6	0.73
QUICKI	0.32 ± 0.02	0.32 ± 0.02	0.59	0.32 ± 0.02	0.32 ± 0.02	0.74
Total cholesterol (mg/dL)	156.7 ± 24.2	154.1 ± 23.6	0.49	157.9 ± 21.0	154.9 ± 23.0	0.39
Triglyceride (mg/dL)^*^	143.9 ± 1.7	140.7 ± 1.6	0.77	134.6 ± 1.6	121.9 ± 1.5	0.19
HDL-C (mg/dL)	43.0 ± 9.3	44.7 ± 13.1	0.37	47.1 ± 11.0	48.1 ± 10.9	0.41
LDL-C (mg/dL)	94.1 ± 21.6	90.3 ± 19.9	0.28	93.9 ± 19.9	88.9 ± 20.0	0.10
hsCRP (mg/L)^*^	1.09 ± 2.78	0.93 ± 3.28	0.39	0.90± 2.79	1.15 ± 2.24	0.09
Mean baPWV (cm/sec)	1597.9 ± 239.6	1606.7 ± 259.6	0.77	1621.8 ± 229.8	1592.0 ± 249.3	0.35
Adiponectin (ng/mL)^*^	8.44 ± 1.82	8.53 ± 1.95	0.72	9.17 ± 1.88	9.92 ± 1.93	0.07
IL-6 (pg/mL)^*^	4.13 ± 3.14	3.21 ± 3.34	0.02	4.33 ± 3.28	3.41 ± 2.48	0.74
TNF-α (ng/dL)^*^	6.66 ± 1.32	6.12 ± 1.36	0.07	6.42 ± 1.32	6.36 ± 1.37	0.87
MCP-1 (ng/dL)^*^	546.6 ± 1.43	577.5 ± 1.38	0.32	588.1 ± 1.57	570.4 ± 1.43	0.67
sVCAM-1 (ng/dL)	1227.6 ± 274.0	1225.1 ± 281.4	0.94	1288.7 ± 285.6	1168.2 ± 252.3	0.0003
sICAM-1 (ng/dL)^*^	158.4 ± 1.38	156.3 ± 1.39	0.70	155.2 ± 1.38	144.0 ± 1.35	0.10

*HOMA-IR* homeostasis model assessment of insulin resistance, *QUICKI* quantitative insulin sensitivity check index, *hsCRP* high sensitivity C-reactive protein, *baPWV* brachial-ankle pulse wave velocity, *IL-6* interleukin-6, *TNF-α* tumor necrosis factor-alpha, *MCP-1* monocyte chemotactic protein-1, *sVCAM-1* soluble vascular cellular adhesion molecule-1, *sICAM-1* soluble intercellular adhesion molecule-1.

Data are presented as the mean ± SD.

^*^Geometric mean ± SD, statistical significance was estimated after logarithmic transformation.

*P*-value were obtained using paired *t*-test based on change (follow-up–baseline) in each group.

Comparing the effect of ciolstazol and placebo on the outcomes, cilostazol significantly reduced sVCAM-1 level (mean difference 105.18 ng/dL, 95% CI 10.65 to 199.71) compared with placebo (Table 3). However, there were no differences observed in baPWV, hsCRP, adiponectin, IL-6, TNF-α, MCP-1, and sICAM-1 between the cilostazol and placebo groups.

**Table 3:** Effect of cilostazol vs. placebo on anthropometric and metabolic parameters

	**Mean difference**	**95% CI for the difference**	***P*-value**
BMI (kg/m^2^)	0.29	−0.03 to 0.61	0.08
Waist circumference (cm)	0.88	−0.28 to 2.03	0.13
Systolic blood pressure (mmHg)	0.76	−4.95 to 6.47	0.79
Diastolic blood pressure (mmHg)	3.07	−1.75 to 7.90	0.21
Fasting glucose (mg/dL)	−3.60	−17.04 to 9.84	0.59
HbA1c (%)^*^	−0.01	−0.05 to 0.03	0.53
Fasting insulin (μIU/mL)^*^	0.07	−0.12 to 0.25	0.45
HOMA-IR^*^	0.06	−0.17 to 0.29	0.59
QUICKI	−0.00	−0.01 to 0.01	0.70
Total cholesterol (mg/dL)	−1.21	−11.46 to 9.05	0.81
Triglyceride (mg/dL)^*^	0.05	−0.15 to 0.26	0.61
HDL-C (mg/dL)	0.98	−3.54 to 5.51	0.66
LDL-C (mg/dL)	−0.71	−9.65 to 8.24	0.87
hsCRP (mg/L)^*^	−0.38	−0.84 to 0.09	0.11
Mean baPWV (cm/sec)	31.42	−55.67 to 118.50	0.47
Adiponectin (ng/mL)^*^	−0.07	−0.17 to 0.03	0.16
IL-6 (pg/mL)^*^	−0.18	−0.61 to 0.26	0.38
TNF-α (ng/dL)^*^	−0.08	−0.22 to 0.06	0.27
MCP-1 (ng/dL)^*^	0.08	−0.11 to 0.27	0.41
sVCAM-1 (ng/dL)	105.18	10.65 to 199.71	0.03
sICAM-1 (ng/dL)^*^	0.06	−0.05 to 0.18	0.28

*HOMA-IR* homeostasis model assessment of insulin resistance, *QUICKI*, quantitative insulin sensitivity check index, *hsCRP* high sensitivity C-reactive protein, *baPWV* brachial-ankle pulse wave velocity, *IL-6* interleukin-6, *TNF-α* tumor necrosis factor-alpha, *MCP-1* monocyte chemotactic protein-1, *sVCAM-1* soluble vascular cellular adhesion molecule-1, *sICAM-1* soluble intercellular adhesion molecule-1.

Mean difference = placebo-cilostazol.

^*^Statistical significance was estimated after logarithmic transformation.

## Discussion

To the best of our knowledge, this is the first randomized controlled trial comparing the effect of cilostazol and placebo on arterial stiffness and biochemical markers related to vascular inflammation including vascular cellular adhesion molecules in diabetic patients without established CVD. Cilostazol treatment for 8 weeks did not substantially change PWV compared with placebo in patients with type 2 diabetes and metabolic syndrome. However, there was significant reduction of serum sVCAM-1 level, and modest improvement of serum adiponectin level with cilostazol treatment.

Arterial stiffness primarily represents elastic property of arteries and has a role in the development of CVD. Various simple and noninvasive methods measuring arterial stiffness have been suggested and PWV is the most widely-used method in the clinical setting. Among various methods of measurement of PWV, baPWV correlates well with carotid-femoral PWV and aortic PWV [20,21], and has been closely associated with incident hypertension [22], insulin resistance [23], and coronary atherosclerosis [24].

Initially, we hypothesized that cilostazol would improve arterial stiffness. Cilostazol is a phosphodiesterase 3 inhibitor; this inhibition and associated increase in cAMP in platelets and vascular smooth muscle cells contributes to the antiplatelet and vasodilatory effects of cilostazol [25]. In several clinical trials, cilostazol treatment attenuated the increase of carotid IMT compared with placebo group in type 2 diabetic patients [13,26]. Recently, the Diabetic Atherosclerosis Prevention by Cilostazol study found that cilostazol more potently inhibited progression of carotid IMT in diabetic patients, even compared with aspirin [12]. In addition, cilostazol increased mean ankle blood flow in patients with arteriosclerosis obliterans [27], and increased coronary flow reserve in patients with coronary artery disease [28].

The present results did not prove the efficacy of cilostazol to improve the arterial stiffness. Although the mean baPWV was slightly decreased with cilostazol and increased with placebo, there was no significant difference between the two groups. There are several possible explanations for this result. First, the study period might not have been long enough. Although there is no established study period for which a clinical outcome becomes apparent, the present study was a relatively short study period compared with other studies involving cilostazol. Second, most of the patients (31 of 37 subjects; 83.7%) had already undergone treatment with angiotensin-converting enzyme inhibitor or angiotensin II receptor blockade, and a considerable number of participants already had taken statins (14 of 37 subjects; 37.8%) because we wanted to investigate the additional effect of cilostazol in a real clinical setting. So, it is possible that further improvement with cilostazol treatment might not be noted due to these drugs that might already affect arterial stiffness. Indeed, in a subgroup analysis, except for the subjects using statins, the effect of cilostazol was more prominent, although not statistically significant (data not shown).

The antiatherogenic and antiimflammatory effects of cilostazol may be come from cAMP-mediated increase in lipoprotein lipase and inhibition of cytokine production. An in vivo study reported cilostazol-associated increase in cardiac lipoprotein lipase in streptozotocin-induced diabetic rats [11]. Cilostazol also significantly reduced the increased TNF-α production induced by lipopolysaccharide in human umbilical vein endothelial cells [29]. In addition, recent in vitro studies found that cilostazol reduced the expression of VCAM-1 and MCP-1 and activate the production of nitric oxide [30,31]. In clinical studies, cilostazol treatment decreased P2Y_12_ reactivity index as a marker of platelet function [32], also decreased hsCRP and soluble CD40 ligand levels, and increased adiponectin level in diabetic patients with peripheral arterial occlusion disease [14].

This study is the first clinical study that proved the beneficial effect of cilostazol on sVCAM-1 level, and adds to the evidence to the clinically-apparent antiinflammatory role of cilostazol. VCAM-1 and ICAM-1 belong to the immunoglobulin gene superfamily and function as endothelial ligands for integrins. Previous studies suggested that VCAM-1 has a role in the recruitment of leukocytes in early atherosclerotic lesions via interaction with VLA-4 integrins on monocytes and lymphocytes [33,34]. Higher levels of soluble VCAM-1 have been documented in subjects with type 2 diabetes [35], metabolic syndrome [36], and atherosclerosis [37], and have been associated with increased CVD mortality [4]. A clinical study showed reduced sVCAM-1 levels in obese women after weight loss over 1-year [38]. Presently, cilostazol modestly decreased IL-6, MCP-1, and sICAM-1 levels in addition to sVCAM-1 with short term treatment. These findings indicate that cilostazol might have an anti-inflammatory role in the early phase of atherosclerosis progression, and it is conceivable that cilostazol could improve other inflammatory markers with a longer treatment period.

Several limitations of our study must be acknowledged. First, this study was confined to type 2 diabetic patients with metabolic syndrome, and results from this study should not be extrapolated to other patient populations. Secondly, our study was performed using a small number of patients over a short period of time. Whether the observed effect of cilostazol on serum sVCAM-1 level in these patients translates into cardiovascular outcomes should be evaluated by a long-term, large scale, randomized, multicenter study. Third, we did not measure other markers related to platelet function or platelet activation such as soluble CD40 ligand, which might be helpful for assessment and interpretation of the effects of cilostazol on vascular inflammation.

## Conclusion

Despite of its potential favorable effects on arterial stiffness, treatment with cilostazol for 8 weeks did not improve the mean baPWV compared with placebo treatment in diabetic patients with metabolic syndrome but not having established CVD. However, this short term treatment was associated with significant reduction of sVCAM-1 level, and modest improvement of adiponectin level. We could not exclude the possibility that cilostazol would improve the vascular outcomes over a longer time. Further studies are needed to elucidate the distinct effects of cilostazol in type 2 diabetes, especially with long-term treatment.

## Competing interests

The authors declare that they have no competing interests.

## Authors’ contributions

NHK and HYK wrote the manuscript and researched data. HA contributed to the data analysis. SGK researched data, reviewed and edited the manuscript. JAS, NHK, KMC, SHB, and DSC contributed to the discussion and reviewed the manuscript. All authors read and approved the final manuscript.

## References

[B1] Fernandez-Real JM Ricart W Insulin resistance and chronic cardiovascular inflammatory syndrome Endocr Rev 2003 24 3 278 301 12788800 10.1210/er.2002-0010

[B2] Goldberg RB Cytokine and cytokine-like inflammation markers, endothelial dysfunction, and imbalanced coagulation in development of diabetes and its complications J Clin Endocrinol Metab 2009 94 9 3171 3182 10.1210/jc.2008-2534 19509100

[B3] Soinio M Marniemi J Laakso M Lehto S Ronnemaa T High-sensitivity C-reactive protein and coronary heart disease mortality in patients with type 2 diabetes: a 7-year follow-up study Diabetes Care 2006 29 2 329 333 10.2337/diacare.29.02.06.dc05-1700 16443882

[B4] de Jager J Dekker JM Kooy A Kostense PJ Nijpels G Heine RJ Bouter LM Stehouwer CD Endothelial dysfunction and low-grade inflammation explain much of the excess cardiovascular mortality in individuals with type 2 diabetes: the Hoorn study Arterioscler Thromb Vasc Biol 2006 26 5 1086 1093 10.1161/01.ATV.0000215951.36219.a4 16514084

[B5] Hayaishi-Okano R Yamasaki Y Katakami N Ohtoshi K Gorogawa S Kuroda A Matsuhisa M Kosugi K Nishikawa N Kajimoto Y Hori M Elevated C-reactive protein associates with early-stage carotid atherosclerosis in young subjects with type 1 diabetes Diabetes Care 2002 25 8 1432 1438 10.2337/diacare.25.8.1432 12145246

[B6] Saremi A Anderson RJ Luo P Moritz TE Schwenke DC Allison M Reaven PD Association between IL-6 and the extent of coronary atherosclerosis in the veterans affairs diabetes trial (VADT) Atherosclerosis 2009 203 2 610 614 10.1016/j.atherosclerosis.2008.07.031 18804762 PMC2688903

[B7] Wadwa RP Noninvasive measures of cardiovascular changes in diabetes mellitus Curr Opin Endocrinol Diabetes Obes 2007 14 4 263 268 10.1097/MED.0b013e32825a674d 17940450

[B8] Cruickshank K Riste L Anderson SG Wright JS Dunn G Gosling RG Aortic pulse-wave velocity and its relationship to mortality in diabetes and glucose intolerance: an integrated index of vascular function? Circulation 2002 106 16 2085 2090 10.1161/01.CIR.0000033824.02722.F7 12379578

[B9] Kambayashi J Liu Y Sun B Shakur Y Yoshitake M Czerwiec F Cilostazol as a unique antithrombotic agent Curr Pharm Des 2003 9 28 2289 2302 10.2174/1381612033453910 14529391

[B10] Lee JH Oh GT Park SY Choi JH Park JG Kim CD Lee WS Rhim BY Shin YW Hong KW Cilostazol reduces atherosclerosis by inhibition of superoxide and tumor necrosis factor-alpha formation in low-density lipoprotein receptor-null mice fed high cholesterol J Pharmacol Exp Ther 2005 313 2 502 509 15734902 10.1124/jpet.104.079780

[B11] Tani T Uehara K Sudo T Marukawa K Yasuda Y Kimura Y Cilostazol, a selective type III phosphodiesterase inhibitor, decreases triglyceride and increases HDL cholesterol levels by increasing lipoprotein lipase activity in rats Atherosclerosis 2000 152 2 299 305 10.1016/S0021-9150(99)00480-3 10998457

[B12] Katakami N Kim YS Kawamori R Yamasaki Y The phosphodiesterase inhibitor cilostazol induces regression of carotid atherosclerosis in subjects with type 2 diabetes mellitus: principal results of the Diabetic Atherosclerosis Prevention by Cilostazol (DAPC) study: a randomized trial Circulation 2010 121 23 2584 2591 10.1161/CIRCULATIONAHA.109.892414 20516379

[B13] Ahn CW Lee HC Park SW Song YD Huh KB Oh SJ Kim YS Choi YK Kim JM Lee TH Decrease in carotid intima media thickness after 1 year of cilostazol treatment in patients with type 2 diabetes mellitus Diabetes Res Clin Pract 2001 52 1 45 53 10.1016/S0168-8227(00)00235-7 11182215

[B14] Hsieh CJ Wang PW Effect of cilostazol treatment on adiponectin and soluble CD40 ligand levels in diabetic patients with peripheral arterial occlusion disease Circ J 2009 73 5 948 954 10.1253/circj.CJ-08-0905 19282610

[B15] O'Donnell ME Badger SA Sharif MA Makar RR Young IS Lee B Soong CV The vascular and biochemical effects of cilostazol in diabetic patients with peripheral arterial disease Vasc Endovascular Surg 2009 43 2 132 143 10.1177/1538574408328586 19131370

[B16] Genuth S Alberti KG Bennett P Buse J Defronzo R Kahn R Kitzmiller J Knowler WC Lebovitz H Lernmark A Follow-up report on the diagnosis of diabetes mellitus Diabetes Care 2003 26 11 3160 3167 14578255 10.2337/diacare.26.11.3160

[B17] Executive summary of the third report of the national cholesterol education program (NCEP) expert panel on detection, evaluation, and treatment of high blood cholesterol in adults (adult treatment panel III) JAMA 2001 285 19 2486 2497 10.1001/jama.285.19.2486 11368702

[B18] Nakamura T Matsuda T Kawagoe Y Ogawa H Takahashi Y Sekizuka K Koide H Effect of pioglitazone on carotid intima-media thickness and arterial stiffness in type 2 diabetic nephropathy patients Metabolism 2004 53 10 1382 1386 10.1016/j.metabol.2004.05.013 15375799

[B19] Uchida H Nakamura Y Kaihara M Sugimoto T Norii H Sasaki M Sato H Makino H Practical efficacy of telmisartan for decreasing morning home blood pressure and pulse wave velocity in patients with mild-to-moderate hypertension Hypertens Res 2004 27 8 545 550 10.1291/hypres.27.545 15492473

[B20] Tanaka H Munakata M Kawano Y Ohishi M Shoji T Sugawara J Tomiyama H Yamashina A Yasuda H Sawayama T Ozawa T Comparison between carotid-femoral and brachial-ankle pulse wave velocity as measures of arterial stiffness J Hypertens 2009 27 10 2022 2027 10.1097/HJH.0b013e32832e94e7 19550355

[B21] Tsuchikura S Shoji T Kimoto E Shinohara K Hatsuda S Koyama H Emoto M Nishizawa Y Brachial-ankle pulse wave velocity as an index of central arterial stiffness J Atheroscler Thromb 2010 17 6 658 665 10.5551/jat.3616 20467192

[B22] Takase H Dohi Y Toriyama T Okado T Tanaka S Sonoda H Sato K Kimura G Brachial-ankle pulse wave velocity predicts increase in blood pressure and onset of hypertension Am J Hypertens 2011 24 6 667 673 10.1038/ajh.2011.19 21331056

[B23] Ho CT Lin CC Hsu HS Liu CS Davidson LE Li TC Li CI Lin WY Arterial stiffness is strongly associated with insulin resistance in Chinese–a population-based study (Taichung Community Health Study, TCHS) J Atheroscler Thromb 2011 18 2 122 130 10.5551/jat.5686 21048381

[B24] Liu CS Li CI Shih CM Lin WY Lin CH Lai SW Li TC Lin CC Arterial stiffness measured as pulse wave velocity is highly correlated with coronary atherosclerosis in asymptomatic patients J Atheroscler Thromb 2011 18 8 652 658 10.5551/jat.7021 21467807

[B25] Ikeda Y Antiplatelet therapy using cilostazol, a specific PDE3 inhibitor Thromb Haemost 1999 82 2 435 438 10605734

[B26] Mitsuhashi N Tanaka Y Kubo S Ogawa S Hayashi C Uchino H Shimizu T Watada H Kawasumi M Onuma T Kawamori R Effect of cilostazol, a phosphodiesterase inhibitor, on carotid IMT in Japanese type 2 diabetic patients Endocr J 2004 51 16 545 550 15644572 10.1507/endocrj.51.545

[B27] Yasuda K Sakuma M Tanabe T Hemodynamic effect of cilostazol on increasing peripheral blood flow in arteriosclerosis obliterans Arzneimittelforschung 1985 35 7A 1198 1200 4074434

[B28] Tanaka T Muneyuki T Oka Y Sada T Kira Y [Effect of long-term cilostazol administration on coronary flow velocity and coronary flow reserve] J Cardiol 1999 34 4 183 188 10553534

[B29] Kim KY Shin HK Choi JM Hong KW Inhibition of lipopolysaccharide-induced apoptosis by cilostazol in human umbilical vein endothelial cells J Pharmacol Exp Ther 2002 300 2 709 715 10.1124/jpet.300.2.709 11805237

[B30] Otsuki M Saito H Xu X Sumitani S Kouhara H Kurabayashi M Kasayama S Cilostazol represses vascular cell adhesion molecule-1 gene transcription via inhibiting NF-kappaB binding to its recognition sequence Atherosclerosis 2001 158 1 121 128 10.1016/S0021-9150(01)00431-2 11500182

[B31] Omi H Okayama N Shimizu M Fukutomi T Nakamura A Imaeda K Okouchi M Itoh M Cilostazol inhibits high glucose-mediated endothelial-neutrophil adhesion by decreasing adhesion molecule expression via NO production Microvasc Res 2004 68 2 119 125 10.1016/j.mvr.2004.05.002 15313121

[B32] Angiolillo DJ Capranzano P Goto S Aslam M Desai B Charlton RK Suzuki Y Box LC Shoemaker SB Zenni MM A randomized study assessing the impact of cilostazol on platelet function profiles in patients with diabetes mellitus and coronary artery disease on dual antiplatelet therapy: results of the OPTIMUS-2 study Eur Heart J 2008 29 18 2202 2211 10.1093/eurheartj/ehn287 18567918

[B33] Elices MJ Osborn L Takada Y Crouse C Luhowskyj S Hemler ME Lobb RR VCAM-1 on activated endothelium interacts with the leukocyte integrin VLA-4 at a site distinct from the VLA-4/fibronectin binding site Cell 1990 60 4 577 584 10.1016/0092-8674(90)90661-W 1689216

[B34] Poston RN Haskard DO Coucher JR Gall NP Johnson-Tidey RR Expression of intercellular adhesion molecule-1 in atherosclerotic plaques Am J Pathol 1992 140 3 665 673 1372160 PMC1886152

[B35] Boulbou MS Koukoulis GN Makri ED Petinaki EA Gourgoulianis KI Germenis AE Circulating adhesion molecules levels in type 2 diabetes mellitus and hypertension Int J Cardiol 2005 98 1 39 44 10.1016/j.ijcard.2003.07.037 15676164

[B36] Gomez Rosso L Benitez MB Fornari MC Berardi V Lynch S Schreier L Wikinski R Cuniberti L Brites F Alterations in cell adhesion molecules and other biomarkers of cardiovascular disease in patients with metabolic syndrome Atherosclerosis 2008 199 2 415 423 10.1016/j.atherosclerosis.2007.11.007 18096167

[B37] Peter K Nawroth P Conradt C Nordt T Weiss T Boehme M Wunsch A Allenberg J Kubler W Bode C Circulating vascular cell adhesion molecule-1 correlates with the extent of human atherosclerosis in contrast to circulating intercellular adhesion molecule-1, E-selectin, P-selectin, and thrombomodulin Arterioscler Thromb Vasc Biol 1997 17 3 505 512 10.1161/01.ATV.17.3.505 9102169

[B38] Ziccardi P Nappo F Giugliano G Esposito K Marfella R Cioffi M D'Andrea F Molinari AM Giugliano D Reduction of inflammatory cytokine concentrations and improvement of endothelial functions in obese women after weight loss over one year Circulation 2002 105 7 804 809 10.1161/hc0702.104279 11854119

